# Update: Influenza Activity — United States, October 1, 2017–February 3, 2018

**DOI:** 10.15585/mmwr.mm6706a1

**Published:** 2018-02-16

**Authors:** Alicia P. Budd, David E. Wentworth, Lenee Blanton, Anwar Isa Abd Elal, Noreen Alabi, John Barnes, Lynnette Brammer, Erin Burns, Charisse N. Cummings, Todd Davis, Brendan Flannery, Alicia M. Fry, Shikha Garg, Rebecca Garten, Larisa Gubareva, Yunho Jang, Krista Kniss, Natalie Kramer, Stephen Lindstrom, Desiree Mustaquim, Alissa O’Halloran, Sonja J. Olsen, Wendy Sessions, Calli Taylor, Xiyan Xu, Vivien G. Dugan, Jacqueline Katz, Daniel Jernigan

**Affiliations:** 1 Influenza Division, National Center for Immunization and Respiratory Diseases, CDC.

Influenza activity in the United States began to increase in early November 2017 and rose sharply from December through February 3, 2018; elevated influenza activity is expected to continue for several more weeks. Influenza A viruses have been most commonly identified, with influenza A(H3N2) viruses predominating, but influenza A(H1N1)pdm09 and influenza B viruses were also reported. This report summarizes U.S. influenza activity* during October 1, 2017–February 3, 2018,^†^ and updates the previous summary (*1*).

## Viral Surveillance

**FIGURE 1:**
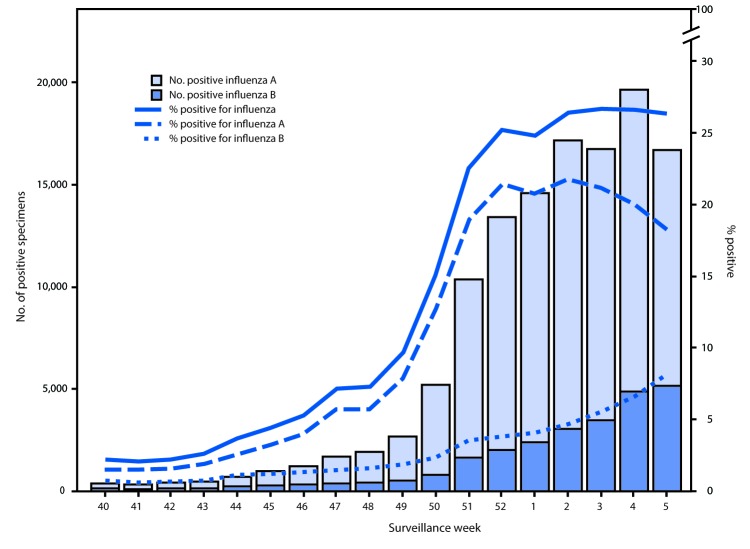
Number* and percentage of respiratory specimens testing positive for influenza reported by clinical laboratories, by influenza virus type and surveillance week — United States, October 1, 2017–February 3, 2018**^†^** * A total of 124,316 (18.7%) of 666,493 specimens tested were positive during October 1, 2017–February 3, 2018. ^†^ As of February 9, 2018.

U.S. World Health Organization (WHO) and National Respiratory and Enteric Virus Surveillance System laboratories, which include both public health and clinical laboratories throughout the 50 U.S. states, Puerto Rico, and the District of Columbia, contribute to virologic surveillance for influenza. During October 1, 2017–February 3, 2018, clinical laboratories tested 666,493 specimens for influenza virus, 124,316 (18.7%) of which tested positive (Figure 1). During this period, the percentage of specimens testing positive for any influenza virus increased to 26.4% during the week ending January 13 and remained at approximately that level (26.3%–26.7%) through the week ending February 3, 2018. The percentage of specimens testing positive for influenza A viruses peaked at 21.8% during the week ending January 13; however, the percentage testing positive for influenza B viruses continued to increase through the week ending February 3, during which 8.1% of specimens tested were positive for influenza B. On a regional level, the percentage of specimens testing positive for any influenza virus has decreased for 2 or more consecutive weeks in U.S. Department of Health and Human Services (HHS) Regions^§^ 6, 7, 9, and 10 but has continued to increase or remain level in the remaining regions (Regions 1, 2, 3, 4, 5, and 8) through the week ending February 3.

**FIGURE 2:**
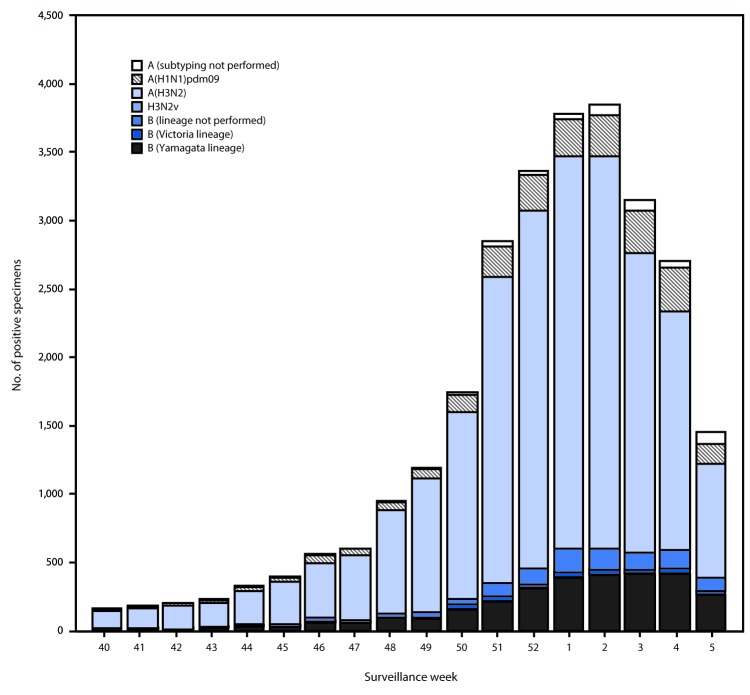
Number* of respiratory specimens testing positive for influenza reported by public health laboratories, by influenza virus type, subtype/lineage, and surveillance week — United States, October 1, 2017–February 3, 2018**^†^** * N = 27,669. ^†^ As of February 9, 2018.

Public health laboratories tested 51,014 specimens collected during October 1, 2017–February 3, 2018. Among these, 27,669 tested positive for influenza virus, including 23,257 (84.1%) for influenza A and 4,412 (15.9%) for influenza B viruses (Figure 2). Among the 22,810 seasonal influenza A viruses subtyped, 20,512 (89.9%) were influenza A(H3N2) viruses, and 2,298 (10.1%) were influenza A(H1N1)pdm09 viruses; influenza A(H3N2) viruses accounted for 74.1% of all influenza viruses reported. Influenza B virus lineage information was available for 3,319 (75.2%) influenza B viruses; 3,010 (90.7%) belonged to the B/Yamagata lineage and 309 (9.3%) to the B/Victoria lineage. Whereas influenza A(H3N2) viruses accounted for the majority of circulating viruses in all HHS regions, the proportion of subtyped influenza A viruses that were identified as A(H1N1)pdm09 regionally ranged from 5% (Region 7) to 21% (Region 6), and the proportion of circulating viruses reported to be influenza B ranged from 9% (Region 5) to 28% (Region 10).

Data on age were available for 23,578 influenza-positive patients whose specimens were tested by public health laboratories. Overall, 1,863 (7.9%) were aged 0–4 years, 5,208 (22.1%) were aged 5–24 years, 7,576 (32.1%) were aged 25–64 years, and 8,931 (37.9%) were aged ≥65 years. Influenza A(H3N2) viruses were predominant among all age groups, accounting for 68%–72% of viruses identified among persons aged 0–4 years, 5–24 years, and 25–64 years and 84% of viruses reported among persons aged ≥65 years. The largest proportion of reported influenza B virus infections occurred in persons aged 5–24 years; influenza B viruses accounted for 21.9% of the viruses reported in this age group.

## Novel Influenza A Viruses

Six human infections with novel influenza A viruses were reported to CDC during October 1, 2017–February 3, 2018. All of these were variant^¶^ virus infections (human infections with influenza viruses that normally circulate in swine). Five of these infections were previously described (*1*). The sixth human infection with a novel influenza A virus was caused by an influenza A(H3N2) variant (A[H3N2]v) virus in Iowa in an adult patient with onset of respiratory symptoms in November 2017. This patient reported exposure to swine during the week preceding illness onset, was not hospitalized, and has fully recovered. No sustained human-to-human transmission was identified.

The A(H3N2)v virus detected in Iowa had a hemagglutinin (HA) gene segment derived from a seasonal human H3N2 virus that was likely introduced into swine by reverse zoonosis (i.e., human infection of swine) in 2010. This virus was closely related to H3N2 viruses known to circulate in the U.S. swine population (*2*), as well as to variant virus infections detected in Delaware, Maryland, Michigan, Nebraska, North Dakota, Ohio, and Pennsylvania during May–December 2017 (*1*,*2*).

## Antigenic and Genetic Characterization of Influenza Viruses

In the United States, public health laboratories participating in influenza surveillance as WHO collaborating laboratories are asked to submit a subset of influenza-positive respiratory specimens to CDC for virus characterization according to specific guidelines.** CDC characterizes influenza viruses through one or more laboratory tests, including genomic sequencing, antigenic characterization by hemagglutination inhibition (HI), or neutralization assays. Circulating viruses that have been isolated and propagated in mammalian cell culture are evaluated for antigenic similarity to cell culture–propagated reference viruses representing the recommended vaccine components of the Northern Hemisphere 2017–18 vaccine.^††^This process is used to assess whether antigenic drift from the vaccine reference viruses has occurred.

All influenza-positive specimens submitted for surveillance and received by CDC are sequenced by next generation sequencing (NGS), using previously described genomic enrichment practices (*3*,*4*) adapted by CDC. NGS uses advanced molecular detection to identify gene sequences from each virus in a sample and thus reveals the genetic variations among many different influenza virus particles in a single sample; these methods also reveal the entire coding region of the genomes. The genomic data are analyzed to determine the genetic identity of circulating viruses and submitted to public databases (GenBank or GISAID EpiFlu). Data obtained from antigenic characterization are important in the assessment of the similarity between reference vaccine viruses and circulating viruses. In vitro antigenic characterization data generated through HI assays or virus neutralization assays are used to assess whether genetic changes in circulating viruses affect antigenicity, which might subsequently affect vaccine effectiveness.

Since the 2014–15 season, many influenza A(H3N2) viruses propagated in tissue culture have lacked sufficient hemagglutination titers for antigenic characterization using HI assays. Therefore, a subset of influenza A(H3N2) viruses are selected for antigenic characterization using the virus neutralization focus reduction assay to assess the ability of various antisera to neutralize infectivity of the test viruses. CDC has antigenically or genetically characterized 1,365 influenza viruses collected and submitted by laboratories in the United States since October 1, 2017, including 276 influenza A(H1N1)pdm09 viruses, 695 influenza A(H3N2) viruses, and 394 influenza B viruses.

**FIGURE 3:**
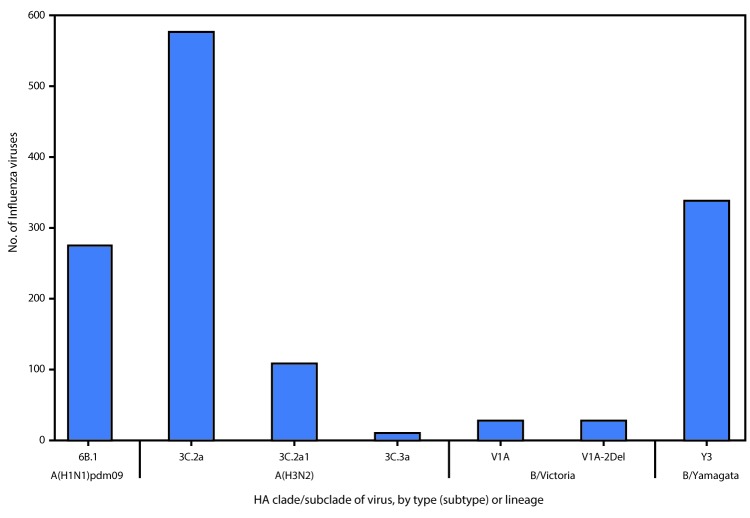
Genetic characterization of U.S. viruses collected during October 1, 2017–February 3, 2018* **Abbreviation:** HA = hemagglutinin. * As of February 9, 2018.

Phylogenetic analysis of the HA gene segments from 276 A(H1N1)pdm09 viruses collected since October 1, 2017, showed that all belonged to subclade 6B.1 (Figure 3). Of the 205 A(H1N1)pdm09 viruses analyzed using HI assays with ferret antisera, 100% were antigenically similar to the cell culture–propagated 6B.1 virus A/Michigan/45/2015, the reference virus representing the A(H1N1)pdm09 vaccine virus for the 2017–18 Northern Hemisphere influenza season.

A total of 695 influenza A(H3N2) viruses were sequenced, and phylogenetic analysis of the HA gene segments illustrated that multiple clades/subclades were cocirculating (Figure 3). Circulating viruses possessed HA gene segments that belonged to clade 3C.2a, subclade 3C.2a1, or clade 3C.3a with 3C.2a predominating (Figure 3). Among the 262 representative A(H3N2) viruses that were antigenically characterized, 257 (98.1%) were well-inhibited (reacting at titers that were within fourfold of the homologous virus titer) by ferret antisera raised against A/Michigan/15/2014 (3C.2a), a cell-propagated A/Hong Kong/4801/2014–like reference virus representing the A(H3N2) component of the 2017–18 Northern Hemisphere influenza vaccines. Although considerable genetic diversity (i.e., multiple cocirculating genetic subgroups) has been observed among the HA gene segments of H3N2 viruses, there have been very few (1.9%) H3N2 viruses showing antigenic drift in the HA this season. In contrast to the 98.1% of viruses that were well-inhibited by ferret antisera raised against cell-propagated A/Michigan/15/2014, only 64.4% of viruses tested were well-inhibited by ferret antiserum raised against the egg-propagated A/Hong Kong/4801/2014 reference virus representing the A(H3N2) vaccine component. This is likely because of egg-adaptive amino acid changes in the HA of the egg-propagated virus. The majority of influenza vaccines used in the United States are produced with egg-based manufacturing.

Among influenza B viruses, phylogenetic analysis of 338 influenza B/Yamagata-lineage viruses showed that all the HA gene segments belonged to clade Y3 (Figure 3). A total of 202 B/Yamagata lineage viruses were antigenically characterized, and all were antigenically similar to cell culture–propagated B/Phuket/3073/2013, the reference virus representing the B/Yamagata-lineage component of quadrivalent vaccines for the 2017–18 Northern Hemisphere influenza season.

Among the 56 influenza B/Victoria-lineage viruses sequenced and phylogenetically analyzed, the HA gene segment of all viruses belonged to genetic clade V1A, the same genetic clade as the vaccine reference virus, B/Brisbane/60/2008. However, the HA gene segment of 28 viruses (50.0%) had a six-nucleotide deletion (encoding amino acids 162 and 163), and viruses like these, abbreviated as V1A-2Del, were previously reported (*5*). Of the 29 influenza B/Victoria viruses that were antigenically characterized, 17 (58.6%) were antigenically similar to cell culture–propagated B/Brisbane/60/2008, the reference virus representing the B/Victoria lineage component of 2017–18 Northern Hemisphere vaccines. All 12 B/Victoria viruses that were poorly inhibited (reacting at titers that were eightfold or more reduced compared with the homologous virus titer) by antisera raised to cell culture–propagated B/Brisbane/60/2008 were V1A-2Del viruses.

## Antiviral Resistance of Influenza Viruses

The WHO Collaborating Center for Surveillance, Epidemiology, and Control of Influenza at CDC tested 1,666 influenza virus specimens collected since October 1, 2017, from the United States for resistance to the influenza neuraminidase inhibitor antiviral medications currently approved for use against seasonal influenza: oseltamivir, peramivir, and zanamivir. Among 376 influenza A(H1N1)pdm09 viruses tested for oseltamivir and peramivir susceptibility, four (1.1%) were resistant to both drugs and contain H275Y, the established NA marker of resistance to oseltamivir. A total of 265 of those influenza A (H1N1)pdm09 viruses also were tested for zanamivir susceptibility, and all were susceptible. All 903 influenza A(H3N2) viruses tested for oseltamivir and zanamivir susceptibility were susceptible to both of these medications. A total of 638 of those A(H3N2) viruses also were tested for peramivir susceptibility, and all were susceptible. All 387 influenza B viruses tested for oseltamivir, peramivir, and zanamivir susceptibility were sensitive to all three recommended antiviral medications. High levels of resistance to the adamantanes (amantadine and rimantadine) persist among influenza A(H1N1)pdm09 and A(H3N2) viruses. Adamantane drugs are not recommended for use against influenza at this time.

## Outpatient Illness Surveillance

**FIGURE 4:**
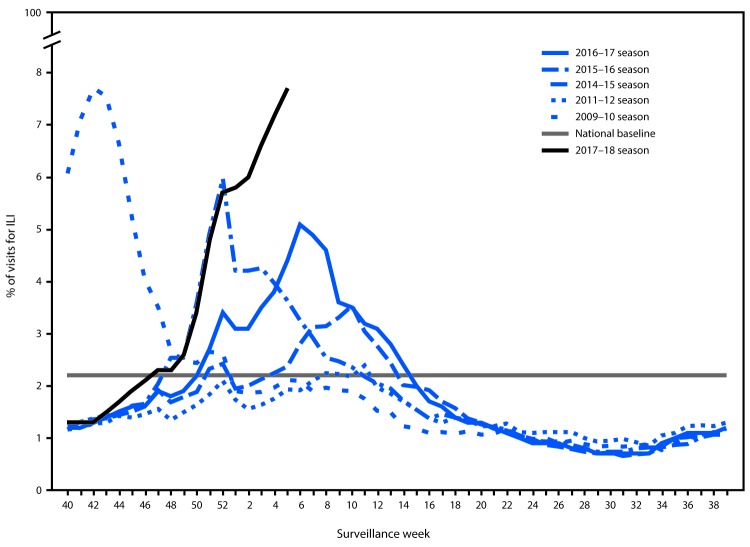
Percentage of outpatient visits for influenza-like illness (ILI)* reported to CDC, by surveillance week — U.S. Outpatient Influenza-Like Illness Surveillance Network (ILINet), 2017–18 influenza season and selected previous influenza seasons^†^ * Defined as fever (temperature of ≥100°F [≥37.8°C], oral or equivalent) and cough or sore throat, without a known cause other than influenza. ^†^ As of February 9, 2018.

During October 1, 2017–February 3, 2018, the weekly percentage of outpatient visits to heath care providers participating in the U.S. Outpatient Influenza-like Illness Surveillance Network (ILINet) for influenza-like illness^§§^ (ILI) ranged from 1.3% to 7.7% (Figure 4). The percentage first exceeded the national baseline^¶¶^ level of 2.2% during the week ending November 25, 2017 (week 47) and has remained at or above the baseline for 11 consecutive weeks so far this season. From the week ending December 23, 2017, (week 51), through the week ending February 3, 2018, (week 5), all 10 HHS regions reported a percentage of outpatient visits for ILI at or above their region-specific baseline levels. ILINet data are also used to produce a weekly jurisdiction-level measure of ILI activity*** ranging from minimal to high. Since the week ending December 30, 2017, more than half of the 53 jurisdictions (50 states, District of Columbia, New York City, and Puerto Rico) experienced high ILI activity each week, with the largest number of jurisdictions (46, 87%) experiencing high ILI activity during the week ending February 3, 2018. During the past five seasons, the largest number of jurisdictions experiencing high ILI activity in a single week ranged from 16 (30%) during the 2015–16 season to 31 (58%) during the 2012–13 and 2014–15 seasons.

## Geographic Spread of Influenza Activity

Influenza activity levels reported by state and territorial epidemiologists indicate the geographic spread of influenza activity^†††^ within their jurisdiction (50 states, District of Columbia, Guam, Puerto Rico, and U.S. Virgin Islands). During the 2017–18 season, the peak number of jurisdictions reporting widespread activity in a single week was 50 (93%); this occurred for the 3 consecutive weeks (weeks ending January 6, January 13, and January 20, 2018). During the previous five influenza seasons, the peak number of jurisdictions reporting widespread activity in a single week during each season has ranged from 41 (76%) in the 2015–16 season to 48 (89%) during the 2012–13 season.

## Influenza-Associated Hospitalizations

CDC monitors hospitalizations associated with laboratory-confirmed influenza infections in adults and children through the Influenza Hospitalization Surveillance Network (FluSurv-NET),^§§§^ which covers approximately 27 million persons (9% of the U.S. population). During October 1, 2017–February 3, 2018, 17,101 laboratory-confirmed influenza-related hospitalizations were reported, representing a cumulative incidence among all age groups of 59.9 per 100,000 population. The hospitalization rate was highest among persons aged ≥65 years, who accounted for 59% of reported influenza-associated hospitalizations.

The cumulative influenza hospitalization rates per 100,000 population during October 1, 2017–February 3, 2018, for persons aged 0–4 years, 5–17 years, 18–49 years, 50–64 years, and ≥65 years were 40.0, 10.3, 18.3, 63.1, and 263.6, respectively. Among all hospitalizations, 14,770 (86.4%) were associated with influenza A virus infection, 2,251 (13.2%) with influenza B virus infection, 43 (0.3%) with influenza A virus and influenza B virus coinfection, and 37 (0.2%) with influenza virus infection for which the type was not determined. Among the 3,841 patients for whom influenza A subtype information was available, 3,308 (86.1%) were infected with influenza A(H3N2) viruses and 533 (13.9%) with influenza A(H1N1)pdm09 viruses. Among hospitalized persons aged 0–64 years for whom influenza A subtype information was available, 23.6% were infected with influenza A(H1N1)pdm09 viruses, compared with only 7.0% of those aged ≥65 years.

Information on underlying medical conditions was available for 2,147 (12.6%) hospitalized patients with laboratory-confirmed influenza as of February 3, 2018. Among 1,955 hospitalized adults with information on underlying medical condition available, 1,325 (67.8%) had at least one underlying medical condition that placed them at high risk for influenza-associated complications. The most commonly reported medical conditions were cardiovascular disease (35.5%), metabolic disorders (33.0%), obesity (25.2%), and chronic lung disease (23.6%). Among 192 hospitalized children with information on underlying medical conditions available, 97 (50.5%) had at least one underlying medical condition, the most commonly reported being asthma (22.8%), neurologic disorders (14.4%), and obesity (10.1%). Among 151 hospitalized women aged 15–44 years with information on pregnancy status, 36 (23.8%) were pregnant.

## Pneumonia and Influenza–Associated Mortality

CDC tracks pneumonia and influenza (P&I)–attributed deaths through the National Center for Health Statistics (NCHS) Mortality Reporting System. The percentages of deaths attributed to P&I are released 2 weeks after the week of death to allow for collection of sufficient data to produce a stable P&I mortality percentage. From October 1, 2017, to January 20, 2018, the weekly percentage of deaths attributed to P&I has ranged from 5.8% to 10.1% and has exceeded the epidemic threshold^¶¶¶^ for 5 consecutive weeks. P&I percentages for recent weeks are likely to be artificially low because of a delay in manual coding for deaths occurring in 2018, and the percentage of deaths caused by P&I is higher among manually coded death certificates than among machine-coded death certificates. The percentage of deaths caused by P&I will likely increase as more data become available.

## Influenza-Associated Pediatric Mortality

As of February 3, 2018, (week 5), 63 laboratory-confirmed influenza-associated pediatric deaths occurring during the 2017–18 season were reported to CDC. Fifteen deaths were associated with an influenza A(H1N1)pdm09 virus infection, 16 were associated with an influenza A(H3N2) virus infection, 14 were associated with infection with an influenza A virus for which no subtyping was performed, and 18 were associated with an influenza B virus infection. Since influenza-associated pediatric mortality became a nationally notifiable condition in 2004, the number of influenza-associated pediatric deaths per season has ranged from 37 to 171, excluding the 2009 pandemic, when there were 358 pediatric deaths during April 15, 2009–October 2, 2010. The mean age of the reported pediatric deaths reported this season was 7.4 years (range 2 months to 17 years); 40 (63%) of the children died after admission to the hospital. Among the 56 children with a known medical history, 30 (54%) had at least one underlying medical condition recognized by ACIP as placing them at increased risk for influenza-related complications. Among the 54 children who were eligible for influenza vaccination (≥6 months of age at date of onset) and for whom vaccination status was known, 14 (26%) had received at least 1 dose of influenza vaccine before onset of illness (13 were fully vaccinated according to 2017 ACIP recommendations, and one had received 1 of 2 recommended doses).

## Discussion

Influenza illness this season has been substantial, with some of the highest levels of ILI and hospitalization rates in recent years and elevated activity occurring in most of the country simultaneously. Influenza A(H3N2) is the predominant influenza virus circulating this season. Past A(H3N2) virus–predominant seasons such as the 2012–13 and 2014–15 seasons had increased numbers of influenza related infections, hospitalizations, and deaths compared with A(H1N1)pdm09 virus-predominant seasons, and the 2017–18 season is on track to reach or exceed estimates from those seasons.

The percentage of outpatient visits to doctors’ offices, urgent care centers, and emergency departments that were for ILI rose sharply in late 2017 to 7.7% in early February. This is the highest level of ILI activity since the pandemic in 2009 which peaked at 7.7%. During the previous five influenza seasons, the peak weekly percentages of outpatient visits for ILI ranged from 3.6% to 6.1% and remained above baseline levels for an average of 16 weeks (range = 11–20 weeks). The weekly percentage of outpatient visits for ILI this season has been above the national baseline for 11 weeks, suggesting that influenza activity is likely to continue for several more weeks.

The cumulative hospitalization rate attributed to laboratory-confirmed influenza for the week ending February 3, 2018, (59.9/100,000) exceeded the rate for the same week in 2014–15 (50.9/100,000), an A(H3N2) virus–predominant season categorized as high severity, and is the highest rate observed for this week since the system expanded to include adults during the 2005–06 season. Persons aged ≥65 years account for the majority of cases (59%); however, hospitalization rates for all adult age groups (18–49 years, 50–64 years, and ≥65 years) are higher than those observed during the same week in 2014–15. These hospitalization rates are not adjusted for testing practices, which can vary from season to season; therefore, caution should be used when comparing hospitalization rates across seasons.

P&I-related deaths also rose sharply in the first weeks of 2018, accounting for 10.1% of all deaths recorded on death certificates during the week ending January 20, 2018. It is anticipated that the number of P&I-related deaths will continue to increase for several more weeks and might exceed the peaks in past recent A(H3N2) virus–predominant seasons (11.1% in 2012–13 and 10.8% in 2014–15). Through the week ending January 20, P&I-related mortality has been above the epidemic threshold for 5 consecutive weeks. During the past five seasons, the average number of weeks this indicator was above threshold was 11 (range of 7–15 weeks).

Sixty-three laboratory-confirmed influenza-associated pediatric deaths have been reported to CDC as of February 3, 2018; 46% of these children were otherwise healthy. Among those children who were eligible for vaccination and for whom vaccination status was known, only 14 (26%) had received any influenza vaccine this season before the onset of illness (13 were fully vaccinated, and one had received 1 of 2 recommended doses). In a previous analysis of pediatric deaths with a similar percentage of eligible children vaccinated (26%), influenza vaccination was associated with a 65% reduction in risk for laboratory-confirmed influenza-associated pediatric death (*6*).

With several more weeks of elevated influenza activity anticipated this season, it is too early to assess overall severity of the season. However, estimates of the burden of influenza disease from the 2012–13 and 2014–15 seasons provide an indication of what might be anticipated for the 2017–18 season. CDC estimated that during each of those seasons influenza accounted for as many as 35.6 million illnesses, 16.6 million medically attended visits, 710,000 hospitalizations and 56,000 deaths.****

Interim estimates of 2017–18 season vaccine effectiveness (VE) against influenza A and influenza B virus infection associated with medically attended acute respiratory illness in the United States was 36% (95% confidence interval [CI] = 27%–44%). VE was estimated to be 25% (95% CI = 13%–36%) against illness caused by influenza A(H3N2) virus, 67% (95% CI = 54%–76%) against A(H1N1)pdm09 virus and 42% (95% CI = 25%–56%) against influenza B virus (*7*). During the 2014–15 season, an A(H3N2) virus–predominant season with high severity and low vaccine effectiveness, influenza vaccine was estimated to have prevented millions of illnesses and tens of thousands of influenza-related hospitalizations. With several more weeks of elevated influenza activity expected, an increasing proportion of influenza A(H1N1)pdm09 and influenza B viruses, and the potential to prevent significant illness through influenza vaccination, CDC continues to recommend influenza vaccination at this time.

During influenza seasons with increased severity, influenza antiviral medications are an increasingly important adjunct to vaccination in the treatment of influenza. Three neuraminidase inhibitor antiviral medications are approved and recommended for use in the United States during the 2017–18 influenza season: oral oseltamivir (available as a generic or under the trade name Tamiflu [Genentech, South San Francisco, California]), inhaled zanamivir (Relenza [GlaxoSmithKline, London, England]) and intravenous peramivir (Rapivab [Seqirus, Summit, New Jersey]). Resistance to these medications is not a concern at this time because only four influenza viruses (all A[H1N1]pdm09 viruses) collected in the United States since October 1, 2017, were identified as not being sensitive to oseltamivir and peramivir.

Treatment with neuraminidase inhibitors has been shown to reduce illness duration and severe outcomes of influenza based on evidence from randomized controlled trials, meta-analyses of randomized controlled trials, and observational studies (*8*,*9*). Treatment with influenza antiviral medications initiated as close to the onset of illness as possible is recommended for patients with confirmed or suspected influenza who have severe, complicated, or progressive illness; who require hospitalization; or who are not hospitalized but who are at high risk for developing serious influenza complications. Treatment should not be delayed while waiting for results of testing or even if rapid antigen-detection influenza diagnostic test results are negative. Clinical benefit of antiviral treatment is greatest when treatment begins within 48 hours after symptom onset; however, antiviral treatment initiated later than 48 hours after illness onset can still be beneficial for some patients (*8*,*10*). A CDC health advisory released on December 27, 2017, regarding treatment with antiviral medications is available at https://emergency.cdc.gov/han/han00409.asp.

Influenza surveillance reports for the United States are posted online weekly (https://www.cdc.gov/flu/weekly). Additional information regarding influenza viruses, influenza surveillance, influenza vaccine, influenza antiviral medications, and novel influenza A infections in humans is available online (https://www.cdc.gov/flu).

**Summary**

**What is already known about this topic?**

CDC collects, compiles, and analyzes data on influenza activity year-round in the United States. Timing of influenza activity and predominant circulating influenza viruses vary by season.

**What is added by this report?**

Influenza activity in the United States began to increase in early November 2017 and rose sharply from December through February 3, 2018. Influenza A viruses have been most commonly identified, with influenza A(H3N2) viruses predominating, but influenza A(H1N1)pdm09 and influenza B viruses were also detected. Influenza illness this season has been substantial, with some of the highest levels of influenza-like illness and hospitalization rates in recent years, and elevated activity occurring in most of the country simultaneously. Elevated influenza activity is expected to continue for several more weeks.

**What are the implications for public health practice?**

With several more weeks of elevated influenza activity expected, the increasing proportion of influenza A(H1N1)pdm09 and influenza B viruses, and the potential to prevent significant illness through influenza vaccination, CDC continues to recommend influenza vaccination at this time. In influenza seasons with increased severity, influenza antiviral medications are an increasingly important adjunct to vaccination in the treatment of influenza. Early treatment with neuraminidase inhibitor antiviral medications is recommended for patients with severe, complicated, or progressive influenza illness and those at higher risk for influenza complications, including adults aged ≥65 years who develop influenza symptoms.

## Footnotes

* The CDC influenza surveillance system collects five categories of information from eight data sources: 1) viral surveillance (U.S. World Health Organization collaborating laboratories, the National Respiratory and Enteric Virus Surveillance System, and novel influenza A virus case reporting); 2) outpatient illness surveillance (U.S. Outpatient Influenza-Like Illness Surveillance Network); 3) mortality (National Center for Health Statistics Mortality Surveillance System and influenza-associated pediatric mortality reports); 4) hospitalizations (FluSurv-NET, which includes the Emerging Infections Program and surveillance in three additional states); and 5) summary of the geographic spread of influenza (state and territorial epidemiologist reports). https://www.cdc.gov/flu/weekly/fluactivitysurv.htm.

† Data as of February 9, 2018.

§ The 10 regions include the following jurisdictions. *Region 1*: Connecticut, Maine, Massachusetts, New Hampshire, Rhode Island, and Vermont; *Region 2*: New Jersey, New York, Puerto Rico, and the U.S. Virgin Islands; *Region 3*: Delaware, District of Columbia, Maryland, Pennsylvania, Virginia, and West Virginia; *Region 4*: Alabama, Florida, Georgia, Kentucky, Mississippi, North Carolina, South Carolina, and Tennessee; *Region 5*: Illinois, Indiana, Michigan, Minnesota, Ohio, and Wisconsin; *Region 6*: Arkansas, Louisiana, New Mexico, Oklahoma, and Texas; *Region 7*: Iowa, Kansas, Missouri, and Nebraska; *Region 8*: Colorado, Montana, North Dakota, South Dakota, Utah, and Wyoming; *Region 9*: Arizona, California, Hawaii, Nevada, American Samoa, Commonwealth of the Northern Mariana Islands, Federated States of Micronesia, Guam, Marshall Islands, and Republic of Palau; *Region 10*: Alaska, Idaho, Oregon, and Washington.

¶ Influenza viruses that circulate in swine are called swine influenza viruses when isolated from swine but are called variant influenza viruses when isolated from humans. Seasonal influenza viruses that circulate worldwide in the human population have important antigenic and genetic differences from influenza viruses circulating in swine.

** Association of Public Health Laboratories. Influenza Virologic Surveillance Right Size Roadmap. https://www.aphl.org/AboutAPHL/publications/Documents/ID_July2013_Influenza-Virologic-Surveillance-Right-Size-Roadmap.pdf.

†† 2017–2018 U.S. trivalent influenza vaccines contain an A/Michigan/45/2015 (H1N1)pdm09–like virus, an A/Hong Kong/4801/2014 (H3N2)–like virus and a B/Brisbane/60/2008–like virus (Victoria lineage). Quadrivalent vaccines will include an additional vaccine virus strain, a B/Phuket/3073/2013–like virus (Yamagata lineage).

§§ Defined as a fever (temperature ≥100°F [≥37.8°C], oral or equivalent) and cough or sore throat, without a known cause other than influenza.

¶¶ The national and regional baselines are the mean percentage of visits for ILI during noninfluenza weeks for the previous three seasons plus two standard deviations. Noninfluenza weeks are defined as periods of ≥2 consecutive weeks in which each week accounted for <2% of the season’s total number of specimens that tested positive for influenza in public health laboratories. National and regional percentages of patient visits for ILI are weighted based on state population. Use of the national baseline for regional data is not appropriate.

*** Activity levels are based on the percentage of outpatient visits in a jurisdiction attributed to ILI and are compared with the average percentage of ILI visits that occur during weeks with little or no influenza virus circulation. Activity levels range from minimal, corresponding to ILI activity from outpatient clinics at or below the average, to high, corresponding to ILI activity from outpatient clinics much higher than the average. Because the clinical definition of ILI is nonspecific, not all ILI is caused by influenza; however, when combined with laboratory data, the information on ILI activity provides a clearer picture of influenza activity in the United States.

§§§ FluSurv-NET conducts population-based surveillance for laboratory-confirmed, influenza-associated hospitalizations in children and adolescents aged <18 years (since the 2003–04 influenza season) and adults aged ≥18 years (since the 2005–06 influenza season). The FluSurv-NET covers approximately 70 counties in the 10 Emerging Infections Program states (California, Colorado, Connecticut, Georgia, Maryland, Minnesota, New Mexico, New York, Oregon, and Tennessee) and additional Influenza Hospitalization Surveillance Project (IHSP) states. IHSP began during the 2009–10 season to enhance surveillance during the 2009 H1N1 pandemic. IHSP sites included Idaho, Iowa, Michigan, Oklahoma, and South Dakota during the 2009–10 season; Idaho, Michigan, Ohio, Oklahoma, Rhode Island, and Utah during the 2010–11 season; Michigan, Ohio, Rhode Island, and Utah during the 2011–12 season; Iowa, Michigan, Ohio, Rhode Island, and Utah during the 2012–13 season; and Michigan, Ohio, and Utah during the 2013–14, 2014–15, 2015–16, 2016–17, and 2017–18 seasons. Cumulative unadjusted incidence rates are calculated using CDC’s National Center for Health Statistics population estimates for the counties included in the surveillance catchment area. Laboratory confirmation is dependent on clinician-ordered influenza testing, and testing for influenza often is underused because of the poor reliability of rapid test results and greater reliance on clinical diagnosis for influenza. Therefore, cases identified as part of influenza hospitalization surveillance likely are an underestimation of the actual number of persons hospitalized with influenza.

¶¶¶ The seasonal baseline proportion of P&I deaths is projected using a robust regression procedure, in which a periodic regression model is applied to the observed percentage of deaths from P&I that were reported by the National Center for Health Statistics Mortality Surveillance System during the preceding 5 years. The epidemic threshold is set at 1.645 standard deviations above the seasonal baseline.

**** Estimates of influenza disease burden and burden of disease averted by influenza vaccination can be found at https://www.cdc.gov/flu/about/disease/burden.htm.
